# Arithmetic hip knee angle measurement on long leg radiograph versus computed tomography—inter-observer and intra-observer reliability

**DOI:** 10.1186/s42836-023-00193-4

**Published:** 2023-08-02

**Authors:** Tom Jan Gieroba, Sofia Marasco, Sina Babazadeh, Claudia Di Bella, Dirk van Bavel

**Affiliations:** 1 Department of Orthopaedics, St Vincent’s Hospital Melbourne, Fitzroy, VIC 3065 Australia; 2 Discipline of Orthopaedics and Trauma, Adelaide University, Adelaide, SA 5005 Australia; 3 College of Medicine and Public Health, Flinders University, Bedford Park, SA 5042 Australia; 4 Sportsmed, Stepney, SA 5069 Australia; 5 Australian Orthopaedic Research Group, Kew East, VIC 3102 Australia; 6 Department of Surgery, The University of Melbourne, Parkville, VIC 3010 Australia; 7 Aikenhead Centre for Medical Discovery (ACMD), St Vincent’s Hospital Melbourne, Fitzroy, VIC 3065 Australia; 8 Department of Surgery, Epworth Healthcare, Richmond, VIC 3121 Australia

**Keywords:** Knee, Arithmetic HKA (aHKA), Total knee arthroplasty, Knee alignment, Robotic arthroplasty

## Abstract

**Background:**

Pre-operative alignment is important for knee procedures including total knee arthroplasty (TKA), especially when considering alternative alignments. The arithmetic Hip Knee Angle (aHKA) is a measure of coronal alignment calculated using the medial proximal tibial (MPTA) and lateral distal femoral angles (LDFA). Traditionally, aHKA is measured on long leg radiographs (LLR). This study assesses the reproducibility of aHKA measurement on LLR and robotic-assisted TKA planning CT.

**Methods:**

Sixty-eight TKA patients with pre-operative LLR and planning CTs were included. Three observers measured the LDFA, MPTA and aHKA three times on each modality and intra-observer and inter-observer reliability was calculated. Statistical analysis was undertaken with Pearson’s r and the Bland–Altman test.

**Results:**

Mean intra-observer coefficient of repeatability (COR) for LLR vs. CT: MPTA 3.50° vs. 1.73°, LDFA 2.93° vs. 2.00° and aHKA 2.88° vs. 2.57° for CT. Inter-observer COR for LLR vs. CT: MPTA 2.74° vs. 1.28°, LDFA 2.31° vs. 1.92°, aHKA 3.56° vs. 2.00°. Mean intra-observer Pearson’s r for MPTA was 0.93 for LLR and 0.94 for CT, LDFA 0.90 for LLR and 0.91 for CT and aHKA 0.92 for LLR and 0.94 for CT. Inter-observer Pearson’s r for LLR compared to CT: MPTA 0.93 vs. 0.97, LDFA 0.91 vs. 0.90, aHKA 0.91 and 0.95.

**Conclusion:**

When compared to LLR, CT measurements of MPTA, LDFA and aHKA are more reproducible and have a good correlation with LLR measurement. CT overcomes difficulties with positioning, rotation, habitus and contractures when assessing coronal plane alignment and may obviate the need for LLRs.

## Introduction

Coronal plane alignment is a key part of surgical planning when performing total knee arthroplasty (TKA) and also for periarticular osteotomies [1, 2]. In TKA, several alignment philosophies exist ranging from Mechanical Alignment (MA) through functional alignment to Kinematic Alignment (KA) [3, 4]. Traditionally, MA was favoured to improve implant survivorship [5]. This strategy has been questioned recently most notably by Bellemans et al. who found that 32% of men and 17% of women have a constitutional varus alignment of 3˚ or more. For these patients, a TKA implanted in mechanical alignment would be non-anatomical [5].

Traditionally, the mechanical hip knee angle (mHKA) has been used to quantify coronal alignment, however, the arithmetic Hip Knee Angle (aHKA) has been more recently described [6]. Both are measured on long leg radiographs (LLR). In order to measure aHKA, the medial proximal tibial angle (MPTA) and lateral distal femoral angle (LDFA) are measured separately, and an arithmetic method is subsequently used to calculate the arithmetic hip knee angle (aHKA = MPTA – LDFA). This measure ignores the normal joint line convergence angle of around –0.5°, therefore when the tibial and femoral joint lines are parallel, the aHKA equals the traditionally measured mHKA [7]. The aHKA has been found to be predictive of the patient’s constitutional knee alignment both when comparing arthritic to non-arthritic patients [6] and comparing arthritic knees to the disease-free contralateral knee in patients with unilateral disease [8]. As the aHKA measurement uses only bony landmarks and is independent of the relationship of the tibia to the femur, it is not affected by their spatial relationship and so joint space narrowing, tibiofemoral subluxation or medial ligamentous laxity to not affect the measured angle [6, 9]. It can also be measured on non-weight-bearing Computed Tomography (CT). Conventional CT has previously been shown to correlate well with LLR for measurement of post-operative TKA alignment [10] and it may be more accurate in the setting of positioning difficulties such as hip or knee flexion contracture, hip rotation contracture or large body habitus, all of which can occur in TKA patients [11].

Robotic assistance for TKA can be imageless or image-based. The most commonly used robotic system is CT based [12] requiring a pre-operative CT for planning and surgical execution. LLR is not always taken as part of a standard TKA workup, especially among surgeons who use CT based planning. As such, the aim of the study was to evaluate the inter-observer and intra-observer reliability of the aHKA measurement on LLR and compare it to aHKA measurements on CT. We hypothesise that MPTA, LDFA and aHKA measurements on CT are more reproducible than on LLR and correlate closely with LLR measurements. Therefore, LLRs and CT could be used interchangeably, and the use of CT based planning could obviate the need for dedicated LLRs.

## Materials and methods

**Fig. 1:**
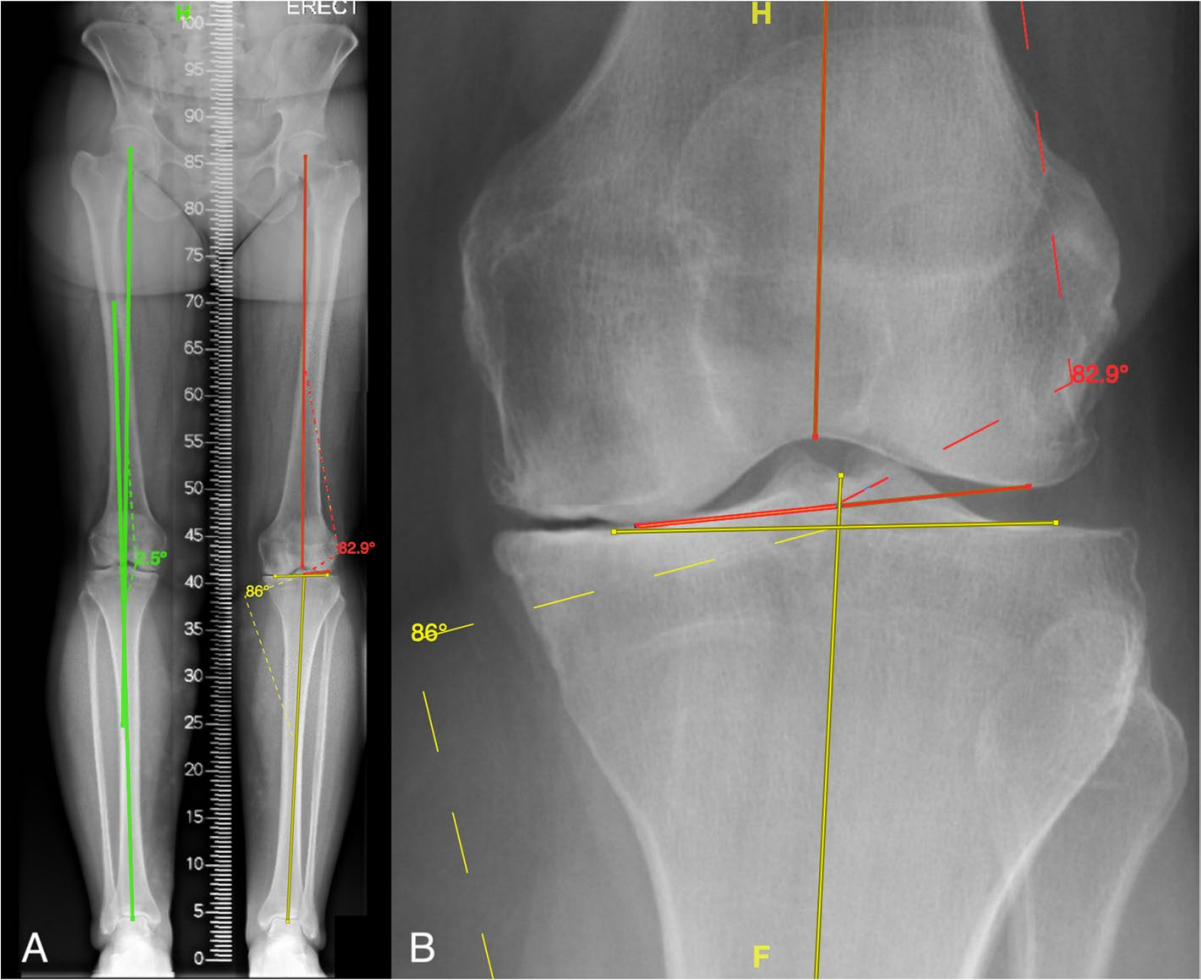
Measuring knee angles on LLR. **a** The mHKA is measured in the right knee (in green). Two lines are drawn—one from the center of the femoral head to the center of the intercondylar notch (representing the mechanical axis of the femur) and another from the center of the tibial plafond to the center of the tibial eminences (representing the mechanical axis of the tibia). The angle subtended by these two lines is the mHKA (2.5°). **b** In the left knee the aHKA is measured. The vertical red line is again the mechanical axis of the femur and the horizontal red line is a line connecting the two most distal articular points of the medial and lateral femoral condyles (representing the femoral joint line). The lateral angle subtended between the two lines is the LDFA (82.9°). The vertical yellow line is the mechanical axis of the tibia, the horizontal yellow line connects the most proximal parts of the medial and lateral tibial plateaus (representing the tibial joint line). The medial angle between the two lines is the MPTA (86.0°). This gives an aHKA of 86.0° – 82.9° = 3.1°

Institutional ethics approval was obtained (HREC 21045). We included patients undergoing robotically assisted TKA at our institution from 2020–2021 using the Stryker Mako (Kalamazoo, MI, USA) surgical robot. At our institution, pre-operative LLR is routinely obtained for patients undergoing TKA and these patients also had Mako planning CT imaging. LLR were obtained with subjects weight-bearing using a set of three 43 cm × 36 cm cassettes with a graduated grid. The lower limbs were fully extended and positioned on a custom-made Perspex footrest that allowed the tibial tuberosities to face forward and the medial malleoli to be 10 cm apart. LLR were measured according to the Paley method [13] using the hospital picture archiving and communication system (PACS) (Centricity Enterprise Web, version 3.0, GE Medical systems, FL, USA). MPTA and LDFA were measured and aHKA was calculated according to the method of Griffiths-Jones et al. [6]. The center of the femoral head was determined using digital templating with concentric circles. The center of the ankle was defined as the midpoint of the tibial plafond. The mechanical axis of the femur was defined as the line from the center of the femoral head to the center of the distal femur at the knee joint. The mechanical axis of the tibia was defined as a line at the midpoint of the tibia at the level of the knee joint to the center of the tibial plafond. The LDFA was defined as the lateral angle between the femoral mechanical axis and the knee joint line of the distal femur. The MPTA was defined as the medial angle formed between the tibial mechanical axis and the knee joint line of the proximal tibia (Fig. 1).

**Fig. 2:**
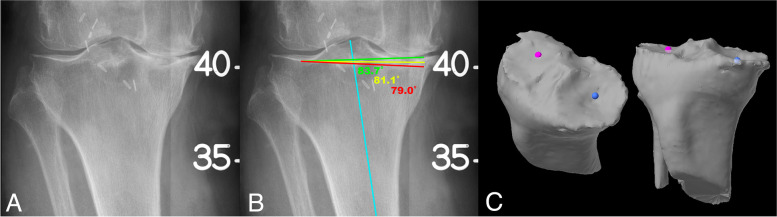
Measuring the tibia. **a** A difficult to measure tibia. **b** The measured MPTA can vary by almost 5° depending on which part of the medial plateau is selected for measurement. **c** 3D CT of the same tibia demonstrating easier determination of the plateau points (2/3 of the way from anterior to posterior in the deepest part of each compartment)

Three observers with varying seniority (senior surgeon (around 500 Mako TKAs), surgical fellow and orthopaedic trainee) performed the measurements at 3 separate sittings 1 week apart. The Mako CTs were performed according to the company protocol with the patient supine and feet-first in the gantry with 3 mm axial slices through the ipsilateral hip, 1 mm slices of the knee joint (including 10 cm proximal and distal) and 3 mm slices through the ipsilateral ankle [14]. The patient remains still for the duration of the scan. A radiolucent rod is attached to the leg to monitor for any motion. These scans are then segmented by company technicians and the bony contours are traced. The scan is used for pre-operative planning of implant positioning and intra-operative adjustment. The same three observers performed measurements on the Mako software (version 1.01) on a dedicated Mako computer in 3 separate sittings 1 week apart. The scans were first correctly patient identified and the segmentation was confirmed. The hip center and malleoli were identified and located in 3 planes. The center of the femur was marked by the most distal point of the intercondylar notch on the axial CT and then confirmed on the coronal and sagittal reconstructions. The center of the tibial plateau was identified as the centre of the tibial eminences. Then, on surface rendered 3 Dimensional (3D) reconstructions, the most distal points of the medial and lateral femoral condyles were selected. On the tibial 3D reconstructions, a point 2/3 of the way from anterior to posterior in the deepest part of the compartment is selected in the medial and lateral tibial condyles (Fig. 2).

**Fig. 3:**
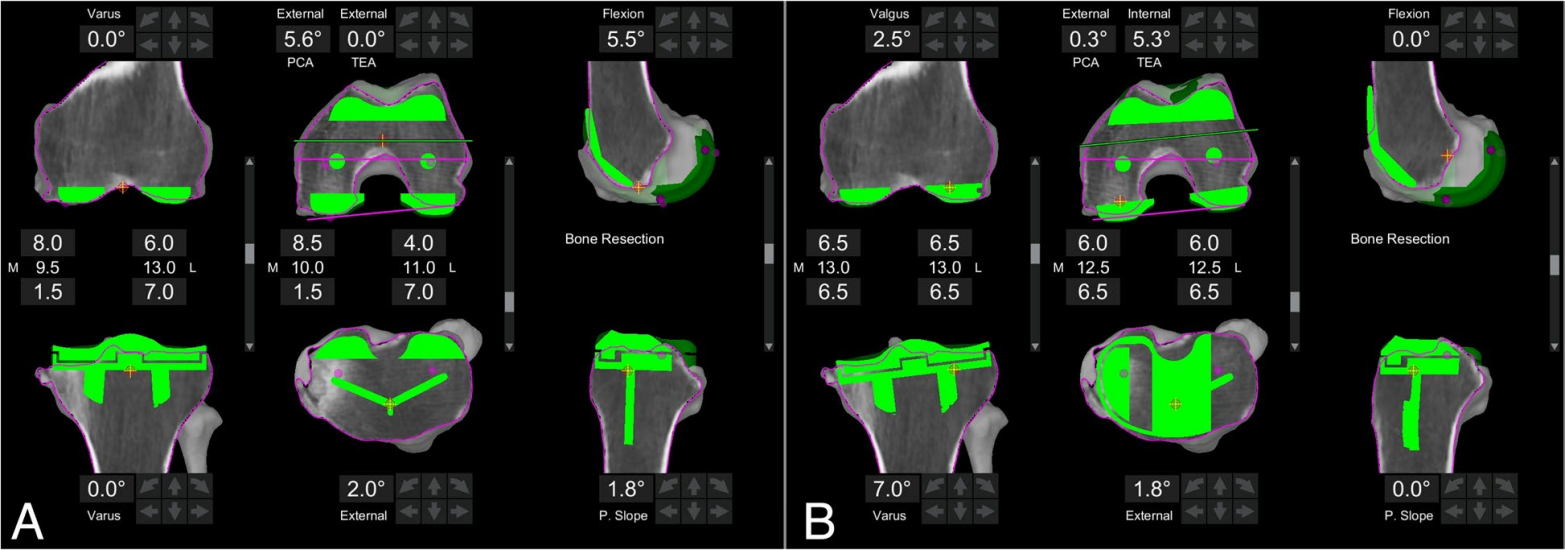
CT measurement. **A** mechanical alignment starting point of TKA component positioning. **B** Components tilted to achieve symmetrical resections (6.5 mm) from distal femur medially and laterally and proximal tibia medially and laterally to give a femoral component valgus of 2.5° (LDFA = 90° – 2.5° = 87.5°) and tibial component varus of 7.0° (MPTA = 90° – 7.0° = 83°) for an overall AHKA of –4.5° or 4.5° varus (MPTA – LDFA = 83° – 87.5°)

Currently, the Mako software does not allow for direct measurement of alignment so to circumvent this limitation the measurements were performed indirectly by virtually positioning a tibial and femoral component in the planning software (Fig. 3) as described by Clark et al. [15]. The tibial component was placed with a 0-degree slope and tilted into varus or valgus until the measured resections from the medial and lateral plateau were equal. The varus angle of the component given by the software subtracted from 90 thus corresponds to the MPTA (valgus tibias are recorded as negative yielding an MPTA of > 90°). The femoral component is placed with 0-degree flexion and tilted into varus/valgus to achieve equal medial and lateral resections from the distal femur. The valgus angle of the component subtracted from 90° thus gives the LDFA (varus femurs are recorded as negative yielding an LDFA of > 90°).

Agreement was assessed with the Pearson Product Moment (r) and coefficient of repeatability (COR). Bland–Altman plots were generated [16, 17]. Pearson’s r ranges from 0 to 1, with values less than 0.5 indicative of poor reliability, values between 0.5 and 0.75 indicating moderate reliability, values between 0.75 and 0.9 indicating good reliability, and values greater than 0.90 indicating excellent reliability [18]. Bland–Altman plots are simple graphic methods for ascertaining agreement between two observers and clearly show which measurements fall outside the limits of agreement. The COR is defined as two standard deviations of the difference between measurements. Ninety-five percent of repeated measurements would be expected to lie within 2 standard deviations of the mean and therefore it is a good indication of repeatability [16]. When comparing CT measures to LLR, the median values of each were compared.

## Results

Sixty-eight patients underwent Mako assisted TKA. Four Mako CTs were not accessible leaving 64 Mako CT scans and 68 LLRs for assessment. Table 1 (LLR) and Table 2 (CT) outline the individual observer measurements for intra-observer reliability (and mean intra-observer values across the 3 observers) as well as the interobserver measurements.

**Table 1:** LLR measurements (degrees)

	**MPTA**				**LDFA**				**aHKA**			
	Max difference	Mean difference	SD	COR	Max difference	Mean difference	SD	COR	Max difference	Mean difference	SD	COR
Observer 1	7.0	1.1	1.19	3.48	5	0.79	0.93	2.65	5.9	1.47	1.2	3.87
Observer 2	8.3	1.2	1.44	4.08	6.9	1.1	1.11	3.32	14.6	1.63	2.2	6.03
Observer 3	4.6	1.06	1.01	3.08	5.1	0.93	0.94	2.81	9.1	1.49	1.62	4.73
Mean intraobserver				3.50				2.93				2.88
Interobserver	6	0.94	0.9	2.74	3.5	0.81	0.75	2.31	6.2	1.34	1.11	3.56

**Table 2:** CT measurements (degrees)

	**MPTA**				**LDFA**				**aHKA**			
	Max difference	Mean difference	SD	COR	Max difference	Mean difference	SD	COR	Max difference	Mean difference	SD	COR
Observer 1	3.9	0.80	0.71	1.42	5.8	0.92	0.95	1.9	6.3	1.33	1.22	2.44
Observer 2	7	0.87	0.85	1.7	7.5	0.76	0.95	1.9	10	1.02	1.28	2.56
Observer 3	8.4	0.62	1.04	2.08	6.0	1.0	1.09	2.18	10.5	1.10	1.35	2.70
Mean intraobserver				1.73				2.00				2.57
Interobserver	3	0.72	0.64	1.28	7.7	0.90	0.96	1.92	7	1.09	1.0	2

### LLR measurement correlation

An excellent intra-observer correlation was found for MPTA (*r* = 0.93, range 0.88–0.93), LDFA (*r* = 0.90, range 0.87–0.92) and aHKA (*r* = 0.92, range 0.87–0.95). Inter-observer correlation was also excellent for MPTA (*r* = 0.93), LDFA (*r* = 0.91) and AHKA (*r* = 0.91).

### CT measurement correlation

An excellent intra-observer correlation was found for MPTA (*r* = 0.94, range 0.91–0.96), LDFA (*r *= 0.91, range 0.87–0.93) and aHKA (*r* = 0.94, range 0.93–0.94). Inter-observer correlation was also excellent for MPTA (*r* = 0.97), LDFA (*r* = 0.90) and AHKA (*r* = 0.95).

### Comparison of LLR and CT

Comparing values obtained on XR and CT, correlation was good. For MPTA *r* = 0.82, for LDFA *r* = 0.87 and for AHKA *r* = 0.85. COR were 2.66°, 2.46° and 3.34°, respectively.

**Fig. 4:**
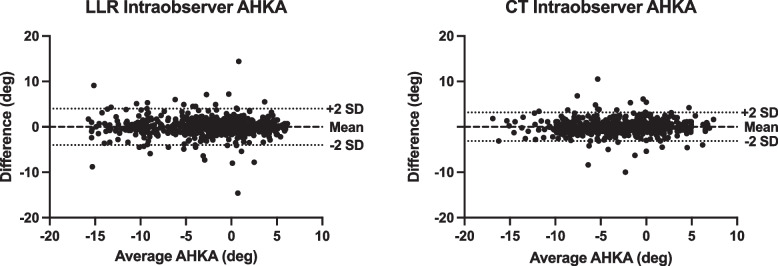
AHKA Intra-observer Bland Altman plot—LLR vs. CT. Both the clustering of points and SDs are tighter on CT measurements

**Fig. 5:**
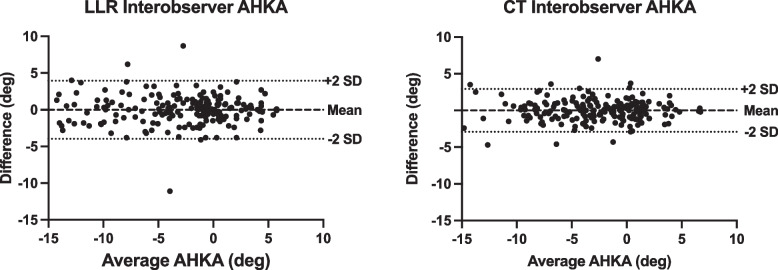
AHKA Inter-observer Bland Altman plot—LLR vs. CT. Both the clustering of points and SDs are tighter on CT measurements

**Fig. 6:**
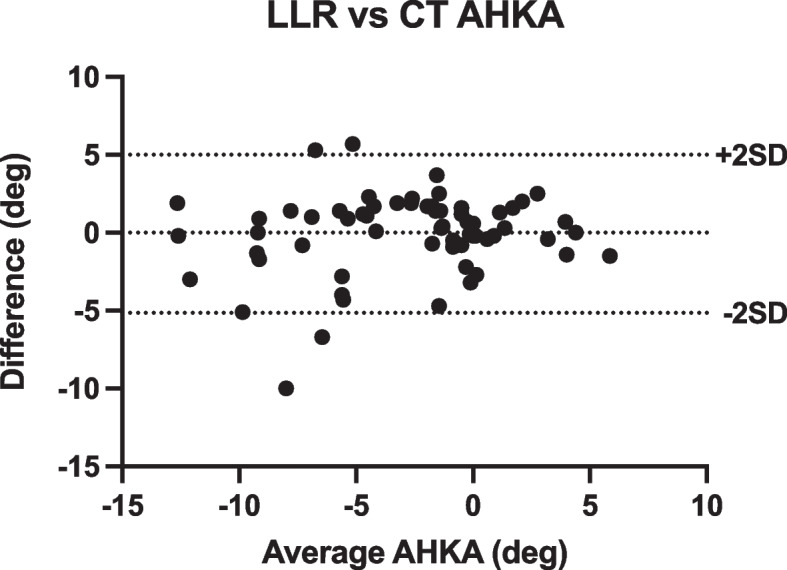
Bland–Altman plot comparing CT to LLR measurements. The median measurement across all trials for each patient are compared

Figures 4, 5 and 6 present Bland–Altman plots for AHKA measurements comparing LLR and CT demonstrating closer correlation, tighter standard deviations and fewer outliers for CT measurements.

## Discussion

This study reveals the intra-observer and inter-observer reliability of measuring MPTA, LDFA and aHKA using LLR and CT and confirms the hypothesis that CT measurements are more reproducible than LLR. Also, there is a good correlation between aHKA measurements on LLR and CT. Of note, there was no significant effect of observer seniority for aHKA measurements on LLR or CT.

Our study has several limitations. All of the included patients had LLR but 4 patients did not have Mako CTs available for measurement. These missing scans may have skewed the results. Also, our technique of angle measurement in the Mako software has been previously described [15] and is applied intra-operatively but has not been validated for its accuracy. Due to the indirect technique of angle measurement on the Mako software (by positioning virtual components), the minimum possible adjustment and smallest measurable difference on the software was 0.5°, but this is below clinical significance and numerically equivalent to the joint line convergence angle which aHKA ignores. This limitation would not be present if the measurement was performed directly on the CT scan. The segmentation and 3D reconstruction of the axial CT scan are performed by the company technicians, and this may affect the identification of surface landmarks when the joint line is being located on the 3D scans (Fig. 2). Finally, our study is purely an imaging study with no clinical correlation. Further research is needed to assess whether more accurate measurement of pre-operative angles improves TKA component positioning or post-operative TKA outcomes.

MacDessi et al. have taken the concept of aHKA further and described a novel classification system; the Coronal Plane Alignment of the Knee (CPAK), which considers the aHKA but also joint line obliquity (JLO = MPTA + LDFA) [7]. Knees can then be subdivided into 9 phenotypes based on the coronal alignment (varus, neutral, valgus) and the JLO (apex distal, neutral, apex proximal). It is useful when planning a TKA especially if the surgeon’s philosophy is to respect the patient’s constitutional alignment. The aHKA and CPAK literature has evaluated the inter and intra-observer variability of the angle measurements on LLR only. Existing literature features fewer observers and fewer rounds of observation [7, 8, 19]. Reported interobserver Pearson’s r ranged from 0.81 to 0.99 and intra-observer Pearson’s r from 0.79 to 0.99. Our assessment of intra and inter-observer reliability with 68 knees, 3 observers and 3 rounds of measurement 1 week apart is the largest assessment of MPTA and LDFA measurement in the literature. Our intra-observer and inter-observer Pearson’s r for LLR measurement of aHKA were 0.92 and 0.91 respectively which are consistent with previous literature. Pearson’s test is useful for assessing linear but not necessarily numerical correlation [20] thus we also performed other statistical analyses. The Bland–Altman test is the gold standard for assessing reproducibility [16, 21].

Two-dimensional CT scanograms have been compared to LLR previously and correlate well however they face similar positioning limitations associated with LLR [10, 22, 23] and these studies have assessed the mHKA rather than the aHKA. One study utilised 3D CT to measure post-operative TKA component position and compared it to LLR and found a Pearson’s r of 0.70 for the femoral component and 0.80 for the tibial component [24]. This study was limited to post-TKA measurements only, had 24 cases and measured implant position rather than overall alignment. Our study is the first in the literature to evaluate pre-operative overall hip knee angle alignment on 3D CT and compare it to LLR and found that it does have good reproducibility.

The LLR is not routinely performed for TKA. It is affected by axial limb rotation and knee flexion contractures [25], whereas CT is not. Using the aHKA rather than mHKA on CT allows a non-weight bearing imaging modality to be used for coronal alignment as aHKA is able to ignore the relationship of the femur to the tibia and uses an arithmetic method rather than a spatial relationship to calculate the angle. CT is an essential part of Mako TKA surgery and is always obtained. The radiation dose of a planning CT is only around 2–3 times more than a complete knee series of radiographs [14]. As the use of robotic assisted TKA increases, the availability of CT for pre-operative coronal plane alignment assessment increases. While no gold-standard modality exists for the assessment of coronal alignment in TKA [10], LLR has been historically used by default. In cases where CT is already required such as with certain robotic systems, CT assessment could become the new default modality replacing LLR for coronal plane alignment measurement. This also applies to other procedures where CT is necessary such as osteotomies around the knee where a CT may be required for the creation of patient specific cutting jigs. From a surgeon's convenience point of view, it reduces the number of different images that need to be accessed and streamlines the process of pre-operative alignment measurement into the workflow of robotic TKA planning. CT based measurement of aHKA may become integrated into robotic TKA planning software in the future. However, where a robotic system does not require a planning CT or for conventional TKA, traditional LLRs remain a valid planning tool especially when using the aHKA which we have shown has excellent inter-observer and intra-observer reliability (Appendix).

## Conclusion

When compared to LLR, CT measurements of MPTA, LDFA and aHKA are more reproducible and have a good correlation with LLR measurement. This finding may indicate that traditional LLR are not required for determination of pre-operative coronal alignment when a CT for planning purposes is undertaken.

## Data Availability

The datasets used and/or analysed during the current study are available from the corresponding author on reasonable request.

## Appendix

A comment on terms; repeatability, reliability and reproducibility: Although sometimes used interchangeably, the terms have specific meanings [26, 27]. In the context of this paper, the term “repeatability” has been used to discuss the “coefficient of repeatability”—a specific statistical tool described by Bland and Altman.

“Reliability” has been used in the context of the whole phrase “intra-observer reliability” or “inter-observer reliability” which assesses the overall accuracy of the measurement, or alternatively, where the term ‘reliability’ has been cited from another paper.

Where “reproducibility” is used, this refers to measurements that were performed by different observers.

## Abbreviations

CT: Computed tomography
LLR: Long leg radiograph
aHKA: Arithmetic hip knee angle
MPTA: Medial proximal tibial angle
LDFA: Lateral distal femoral angle
mHKA: Mechanical hip knee angle
TKA: Total knee arthroplasty
CPAK: Coronal plane alignment of the knee
JLO: Joint line obliquity
COR: Coefficient of repeatability
